# Comparative Efficacy of Different Drugs for Lower Urinary Tract Symptoms due to Benign Prostatic Hyperplasia: A Bayesian Network Meta-Analysis

**DOI:** 10.3389/fphar.2022.763184

**Published:** 2022-03-07

**Authors:** Zhinan Fan, Hongjin Shi, Jinsong Zhang, Haifeng Wang, Jiansong Wang

**Affiliations:** Department of Urology, The Second Affiliated Hospital of Kunming Medical University, Kunming, China

**Keywords:** network meta-analysis, benign prostatic hyperplasia, lower urinary tract symptoms, randomized controlled trial, drug treatment

## Abstract

**Background:** Lower urinary tract symptoms (LUTS) caused by benign prostatic hyperplasia (BPH) are common in middle-aged and elderly men. The current drugs for treating this disease include α1-adrenoceptor antagonists (ABs), muscarinic receptor antagonists (MRAs), phosphodiesterase five inhibitors (PDE5-Is), and β3-adrenoceptor agonists (B3As). However, direct comparative studies analyzing different therapies are limited; therefore, we conducted a network meta-analysis (NMA) to evaluate the efficacy of different drug regimens for treating BPH/LUTS.

**Methods:** The PubMed, EMbase, Web of Science, and Cochrane Library databases were searched to collect randomized controlled trials (RCTs) of different drug treatments for BPH/LUTS from January 2000 to April 2021. The NMA was performed using R 4.1 software.

**Results:** Fifty-five RCTs were included among a total of 1639 trials. ① ABs + PDE5-Is, ABs + B3As, ABs + MRAs, ABs, and PDE5-IS were superior to the placebo in improving the total International Prostate Symptom Score (IPSS), IPSS-Voiding, and IPSS-storage. ② For increasing the maximum flow rate (Qmax), ABs + PDE5-Is, ABs + MRAs, and ABs were more effective than the placebo. ③ Regarding reducing post-void residual urine (PVR), none of the six treatment plans had significant effects.

**Conclusion:** Combination therapy showed greater efficacy than monotherapy, and ABs + PDE5-Is was the most successful treatment for improving the overall IPSS score. ABs are a primary therapeutic measure to increase Qmax, and ABs + PDE5-I may be a more suitable choice for enhancing Qmax. The combination of MRA and AB+ MRA may lead to an increase in PVR.

**Systematic Review Registration**: [website], identifier [registration number].

## 1 Introduction

Benign prostatic hyperplasia (BPH) is a common cause of lower urinary tract symptoms (LUTS) in middle-aged and elderly men. LUTS include obstructive, irritative, and postmicturition symptoms (Li et al., 2014). Recently, the number of people diagnosed with BPH/LUTS has gradually increased due to the extension of men’s life expectancy, increased disease awareness and diagnosis, and the desire to improve the quality of life (Egan, 2016). Recent epidemiological surveys indicate a significant increase in the global incidence of BPH, with the prevalence of this condition increasing by 105.70% from 1990 to 2019. The burden of BPH is concentrated in Asia and Eastern Europe, and its incidence is the highest in the 65–69 age group (Zhu et al., 2021). A large-scale prospective cohort study pointed out that the incidence and progression rate of LUTS are very high and rise sharply with age in men (Platz et al., 2012). BPH/LUTS not only affects patients’ daily life and work but is also associated with erectile dysfunction and psychological disorders in male patients, leading to a decline in their quality of life (Garg et al., 2020). A variety of drugs are available to treat the disease, such as α1-adrenoceptor antagonists (ABs), muscarinic receptor antagonists (MRAs), phosphodiesterase five inhibitors (PDE5-Is), and β3-adrenoceptor agonists (B3As). Related studies have pointed out that the combination of ABs and other drugs can achieve better efficacy. However, direct comparison studies that analyze different therapies are limited to date. Wang et al. (2014) conducted a network meta-analysis (NMA) to comprehensively evaluate the various treatment measures for the first time. The results indicated that AB plus PDE5-Is is the most effective solution for improving the total International Prostate Symptom Score (IPSS) score; however, B3As was not included in this study (Wang et al., 2014). Thus, we conducted an NMA to evaluate the BPH/LUTS treatment efficacy with various single drugs and combinations of drugs and ranked the different treatment options for reference. To expand upon the earlier NMA, our study included more recent literature, particularly B3As-related research.

## 2 Methods

We followed the Preferred Reporting Items for Systematic Reviews and Meta-Analyses (PRISMA) and the extension statement for network meta-analysis (Moher et al., 2009; Hutton et al., 2015).

### 2.1 Inclusion Criteria

Participants: Patients diagnosed with BPH/LUTS.

Interventions and Comparisons: ABs, MRAs, PDE5-Is, ABs plus B3As, ABs plus MRAs, and ABs plus PDE5-Is were used for the intervention, and the control group was administered either a placebo or an appeal drug.

Outcomes: International prostate symptom score (IPSS), IPSS-storage, IPSS-voiding, maximum flow rate (Qmax), and post-void residual urine (PVR).

Study design: Clinical randomized controlled trials (RCTs).

### 2.2 Exclusion Criteria

The exclusion criteria were as follows: 1. non-randomized controlled trials, 2. duplicate documents, and 3. provided a non-English full text.

### 2.3 Search Strategy

English literature on drug treatment for BPH/LUTS published from January 1, 2000 to March 2021 was collected by digitally searching the PubMed, Web of Science, EMbase, and Cochrane Library databases. The keywords used for the search were prostatic hyperplasia, lower urinary tract symptoms, α-blockers, muscarinic receptor antagonists, phosphodiesterase five inhibitors, and β3-adrenoceptor agonists.

### 2.4 Data Extraction

Literature screening and data extraction were performed independently by two researchers according to the inclusion and exclusion criteria. We considered the mean change from baseline to the end of the study, rather than the post-intervention value for each outcome indicator, as an effective measure. Studies with missing information were estimated according to the method reported in the Cochrane Handbook for Systematic Reviews of Interventions (Cumpston et al., 2019). If there was a disagreement, it was resolved through discussion or the assistance of a third researcher, and the following information was extracted from the literature meeting the inclusion criteria: (1) the authors and time of the included documents; (2) sample size, medication type and dosage, and treatment time for the test and control groups; and (3) extraction of various outcome indicators. Finally, the two independent analyses were cross-checked.

### 2.5 Quality Assessment

Two researchers independently assessed the quality of each study using the Cochrane Collaboration tool for assessing the risk of bias (Higgins et al., 2011).

### Statistical Analysis

RevMan5.3 software was used for pairwise meta-analysis. Standardized mean difference was used as the effect analysis statistic for the measurement data, with a 95% confidence interval (CI) for effect sizes. The heterogeneity of the included literature was evaluated using *P*-values and I^2^. If *p* > 0.1 and I^2^ < 50%, indicating no heterogeneity, the fixed-effect model was adopted. Otherwise, the random effect model was adopted. Then, we used R 4.1 software for Bayesian network meta-analysis. All efficacy assessments were performed using random- or fixed-effect models, after which the most suitable model was selected according to the lowest Bayesian deviance information criterion (DIC). If the DIC of the random-effect model decreased by > 5 compared with that of the fixed-effect model, the random-effect model was selected. In addition, the node-splitting method was used to test the local inconsistency of the included studies, and *p* > 0.05 was considered as good consistency. The effects of the intervention were sorted by the surface under the cumulative ranking curve (SUCRA); the larger the SUCRA value, the higher the ranking, indicating that the intervention was more likely to be the most successful intervention. Funnel plots were used to evaluate the publication bias in the included studies. Egger’s test was used to test the asymmetry of the funnel plot. To determine whether the results were affected by the study characteristics, we performed a subgroup network meta-analysis according to whether the treatment time was ≥12 weeks.

## 3 Results

### 3.1 Search Results and Study Characteristics

**FIGURE 1:**
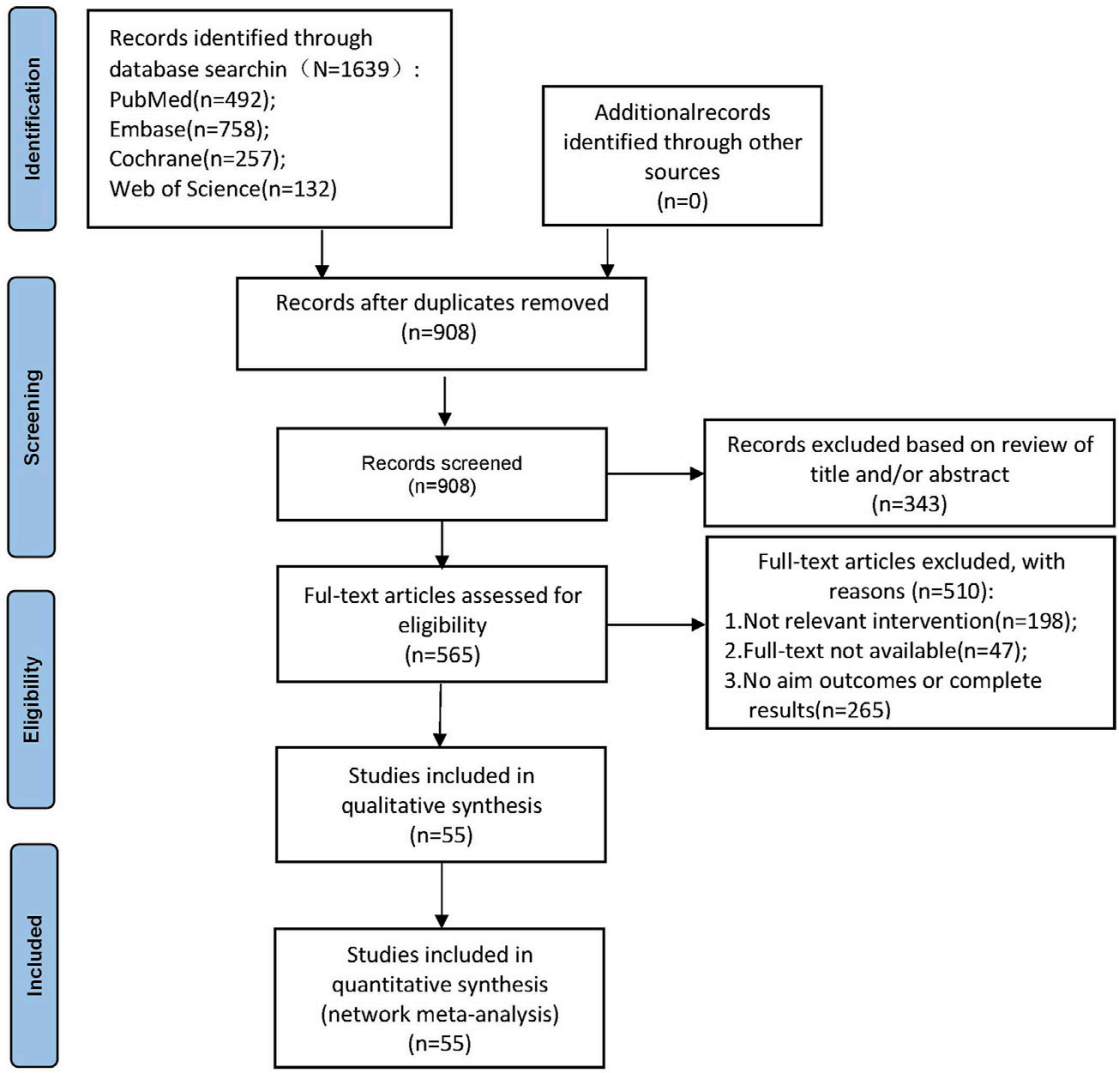
Flow diagram of the identification process for eligible studies.

A total of 1639 related documents were obtained in the initial inspection, and 55 RCTs were finally included after the layer-by-layer screening, including 24,576 patients who were treated with six different drug therapies, namely ABs, MRAs, PDE5-Is, ABs plus B3As, ABs plus MRAs, and ABs plus PDE5-Is. Of the 55 RCTs, nine studies compared the appealing drug with a placebo, 20 studies compared different drugs, and 26 multi-arm studies compared different drugs or with a placebo. The treatment time ranged from 4 weeks to 3 months, of which patients in 41 studies were treated for 12 weeks. The literature screening process and results are shown in Figure 1. The baseline characteristics of the included studies are summarized in Supplementary Table S1.

### 3.2 Quality Assessment

**FIGURE 2:**
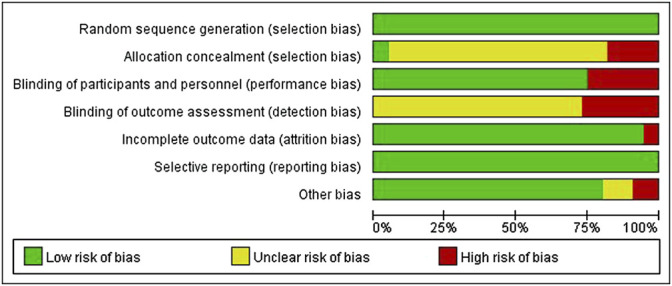
Risk of bias summary plot.

**FIGURE 3:**
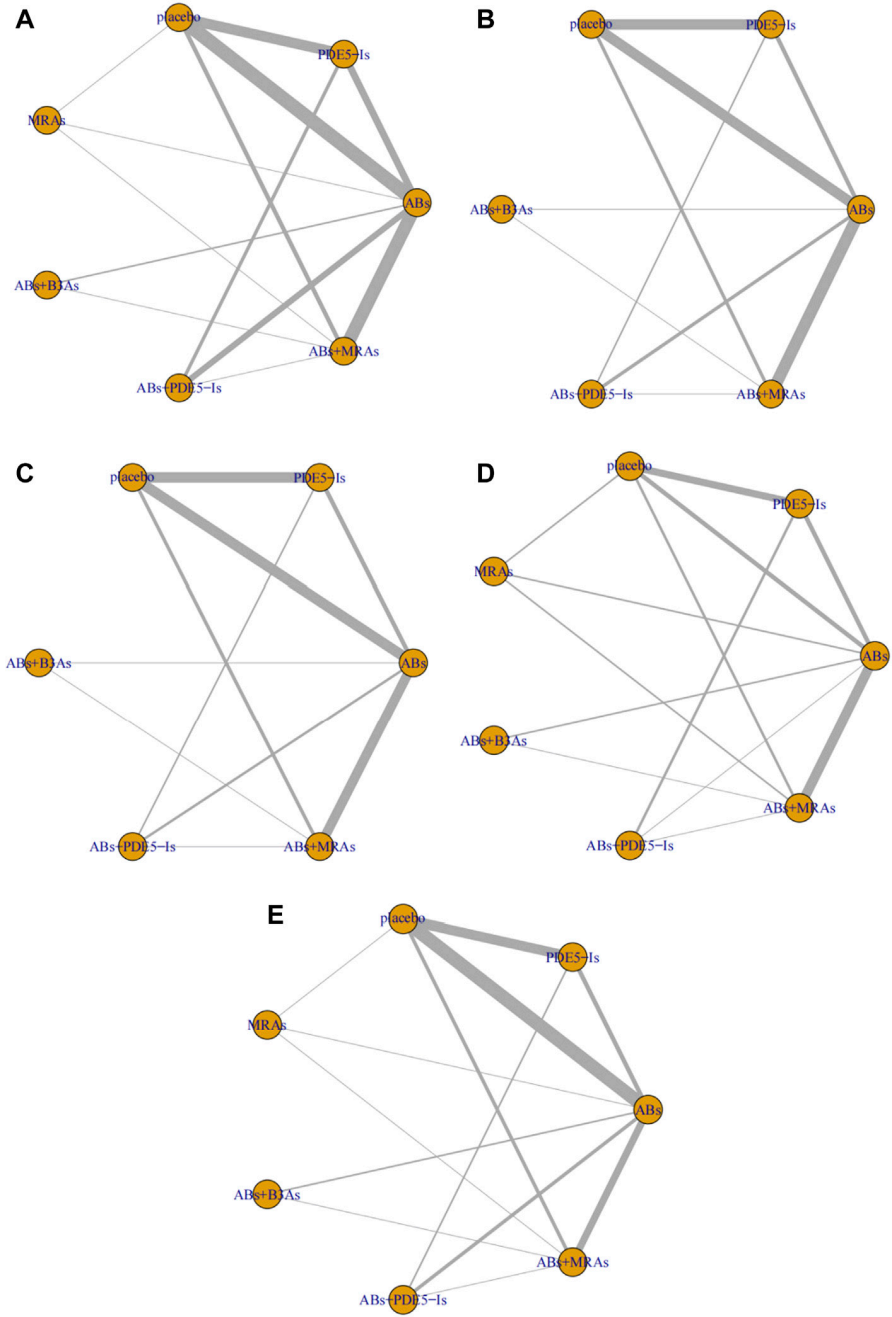
Network plots for different outcomes. **(A)** International Prostate Symptom Score (IPSS); **(B)** IPSS-storage; **(C)** IPSS-voiding; **(D)** post-void residual urine (PVR); **(E)** maximum flow rate (Qmax). include α1-adrenoceptor antagonists (ABs), muscarinic receptor antagonists (MRAs), phosphodiesterase five inhibitors (PDE5-Is), and β3-adrenoceptor agonists (B3As).

All the included studies were RCTs; 42 were blind, 23 were multi-country and multi-center studies, and only three studies provided information about distribution hiding. and no selected the reporting outcomes was found in all studies. As shown in Figures 2, 3 and Supplementary Figure S1.

### 3.3 Pairwise Meta-Analysis

The results of the pairwise meta-analysis and subgroup analysis are detailed in Supplementary Table S2.

#### 3.3.1 IPSS

Compared with that for the placebo, the IPSS significantly decreased under AB, PDE5-I, and ABs + MRA treatment. In addition, ABs + PDE5-Is were more effective than ABs and PDE5-Is alone. No significant change was found in the subgroup analysis according to specific drug types. Further analysis of the sub-scores of IPSS-storage and IPSS-voiding showed that in terms of IPSS-storage, ABs + PDE5-Is and ABs + MRAs were more effective than ABS. In IPSS-voiding, ABs + PDE5-Is still showed an improved curative effect compared to that of ABS; however, the curative effect of ABs + MRAs showed no significant difference from that of ABs.

#### 3.3.2 Qmax

Compared with that of the placebo, ABs had a greater therapeutic effect; however, the subgroup analysis showed no significant difference between tamsulosin and the placebo.

#### 3.3.3 PVR

Compared with that of the placebo, ABs effectively reduced the PVR, while ABs + MRAs increased the PVR. Compared with that of ABs, PDE5-Is, ABs + MRAs, and ABs + B3As significantly increased the PVR.

### 3.4 Network Meta-Analysis

#### 3.4.1 IPSS

**FIGURE 4:**
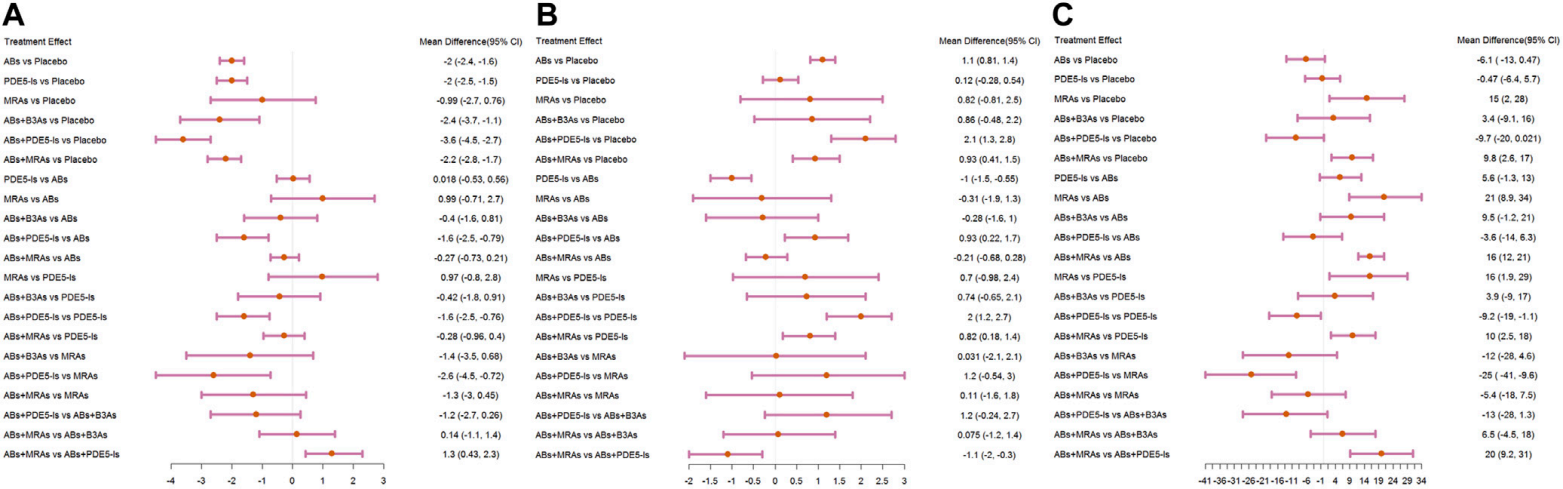
Forest plots. **(A)** International Prostate Symptom Score (IPSS); **(B)** maximum flow rate (Qmax); **(C)** post-void residual urine (PVR). include α1-adrenoceptor antagonists (ABs), muscarinic receptor antagonists (MRAs), phosphodiesterase five inhibitors (PDE5-Is), and β3-adrenoceptor agonists (B3As).

**FIGURE 5:**
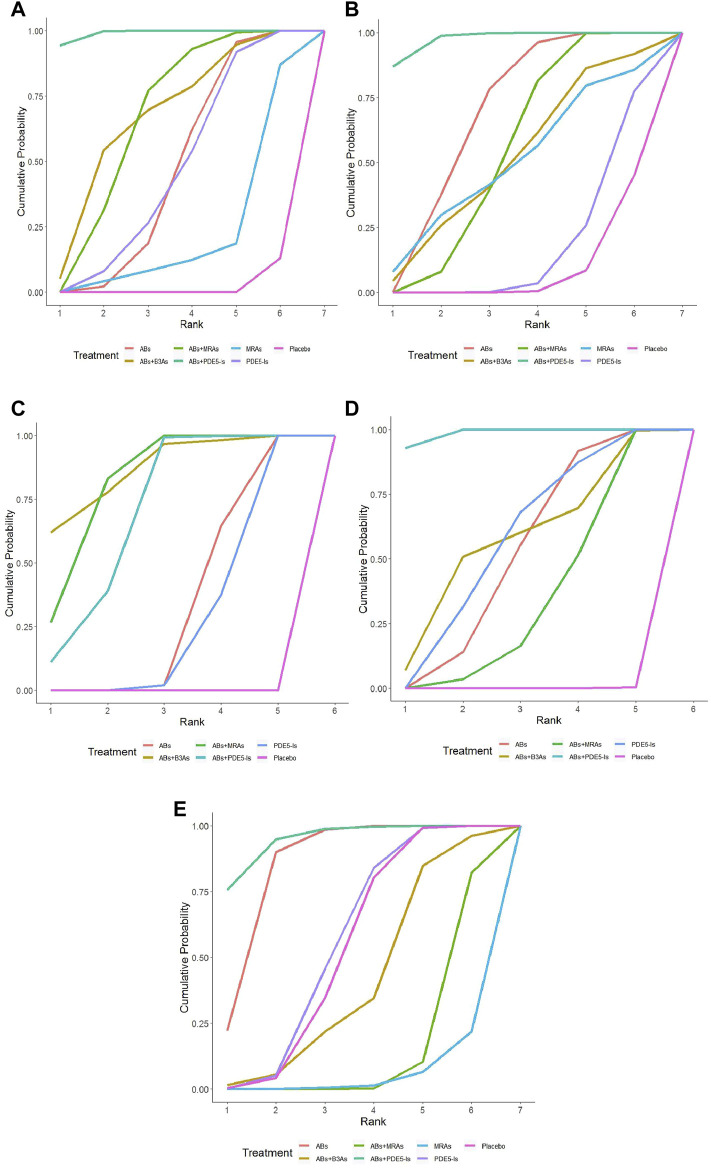
Surface under the cumulative ranking curve (SUCRA) plots. **(A)** International Prostrate Symptom Score (IPSS) SUCRA values: α1-adrenoceptor antagonists (ABs)+ phosphodiesterase five inhibitors (PDE5−Is) (99.02%), ABs+β3-adrenoceptor agonists (B3As) (67.07%), ABs + muscarinic receptor antagonists (MRAs) (66.78%), PDE5−Is (46.69%), ABs (46.47%), and MRAs (21.8%); **(B)** IPSS-storage SUCRA values: ABs + B3As (86.9%), ABs + MRAs (81.97%), ABs + PDE5−Is (69.9%), ABs (33.3%), and PDE5−Is (27.93%); **(C)** IPSS-voiding SUCRA values: ABs + PDE5−Is (98.55%), ABs + B3As (57.48%), PDE5−Is (57.37%), ABs (52.2%), and ABs + MRAs (34.31%); **(D)** Maximum flow rate (Qmax) SUCRA values: ABs + PDE5−Is (97.58%), ABs 68.73%), ABs + MRAs (54.82%), ABs + B3As (51.78%), MRAs (50.2%), and PDE5−Is (17.86%); **(E)** Post-void residual urine (PVR) SUCRA values: ABs + PDE5−Is (94.81%), ABs (85.1%), PDE5−Is (55.67%), ABs + B3As (40.75%), ABs + MRAs (15.49%), and MRAs (5.06%).

**FIGURE 6:**
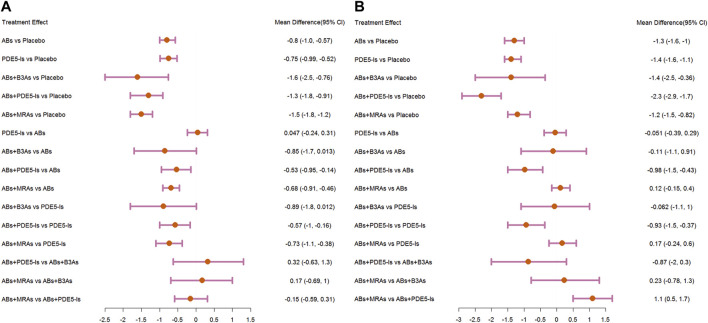
Forest plots. **(A)** International Prostate Symptom Score (IPSS)-storage; **(B)** IPSS-voiding. include α1-adrenoceptor antagonists (ABs), muscarinic receptor antagonists (MRAs), phosphodiesterase five inhibitors (PDE5-Is), and β3-adrenoceptor agonists (B3As).

The IPSS analysis was based on 55 studies, and the resulting network plot is shown in Figure 3A. Compared with that of the placebo, ABs, PDE5-Is, ABs + B3As, ABs + MRAs, and ABs + PDE5-Is effectively reduced the total IPSS score. However, MRA monotherapy showed no improvement, and ABs + PDE5-Is improved the total IPSS relative to that of AB monotherapy (Figure 4A). Based on the SUCRA results, the effective rate was sorted from highest to lowest as ABs + PDE5-Is (99.02%), ABs + B3As (67.07%), ABs + MRAs (66.78%), PDE5-Is (46.69%), ABs (46.47%), and MRAs (21.8%) (Figure 5A). Further analysis of the IPSS-storage and IPSS-voiding sub-scores was completed (Figures 3B,C). The original study in this analysis did not include MRA monotherapy. Compared with those of the placebo, the remaining five treatment regimens effectively reduced the two sub-scores (Figures 6A,B). The order of the SUCRA in the IPSS-storage group was ABs + B3As (86.9%), ABs + MRAs (81.97%), ABs + PDE5-Is (69.9%), ABs (33.3%), and PDE5-Is (27.93%) (Figure 5B). The order of the SUCRA in the IPSS-voiding group was ABs + PDE5-Is (98.55%), ABs + B3As (57.48%), PDE5-Is (57.37%), ABs (52.2%), and ABs + MRAs (34.31%) (Figure 5C). Subgroup analysis was conducted according to the treatment time, and no significant changes were observed in drug sequencing (Supplementary Figures S2–S5).

#### 3.4.2 Qmax

The analysis of Qmax was based on 41 studies, and the network plot is shown in Figure 3E. Compared with that of the placebo, ABs, ABs + PDE5-Is, and ABs + MRAs effectively increased the Qmax (Figure 4B), and ABs + PDE5-Is were more successful than ABs at improving Qmax. According to the SUCRA results, the order of efficiency was ABs + PDE5-Is (97.58%), ABs (68.73%), ABs + MRAs (54.82%), ABs + B3As (51.78%), MRAs (50.2%), and PDE5-Is (17.86%) (Figure 5D). No significant change was found in the subgroup analysis according to the treatment time (Supplementary Figures S6–S9).

#### 3.4.3 PVR

The PVR analysis was based on 28 studies, and the network plot is shown in Figure 3D. Compared with that of the placebo, none of the six treatments effectively reduced the PVR; however, MRA and AB + MRA increased the PVR (Figure 4C). According to the SUCRA results, the order of efficiency was ABs + PDE5−Is (94.81%), ABs (85.1%), PDE5−Is (55.67%), placebo (53.11%), ABs + B3As (40.75%), ABs + MRAs (15.49%), and MRAs (5.06%) (Figure 5E). No significant change was found in the subgroup analysis according to the treatment time (Supplementary Figures S10, S11).

### 3.5 Consistency and Publication Bias Tests

**FIGURE 7:**
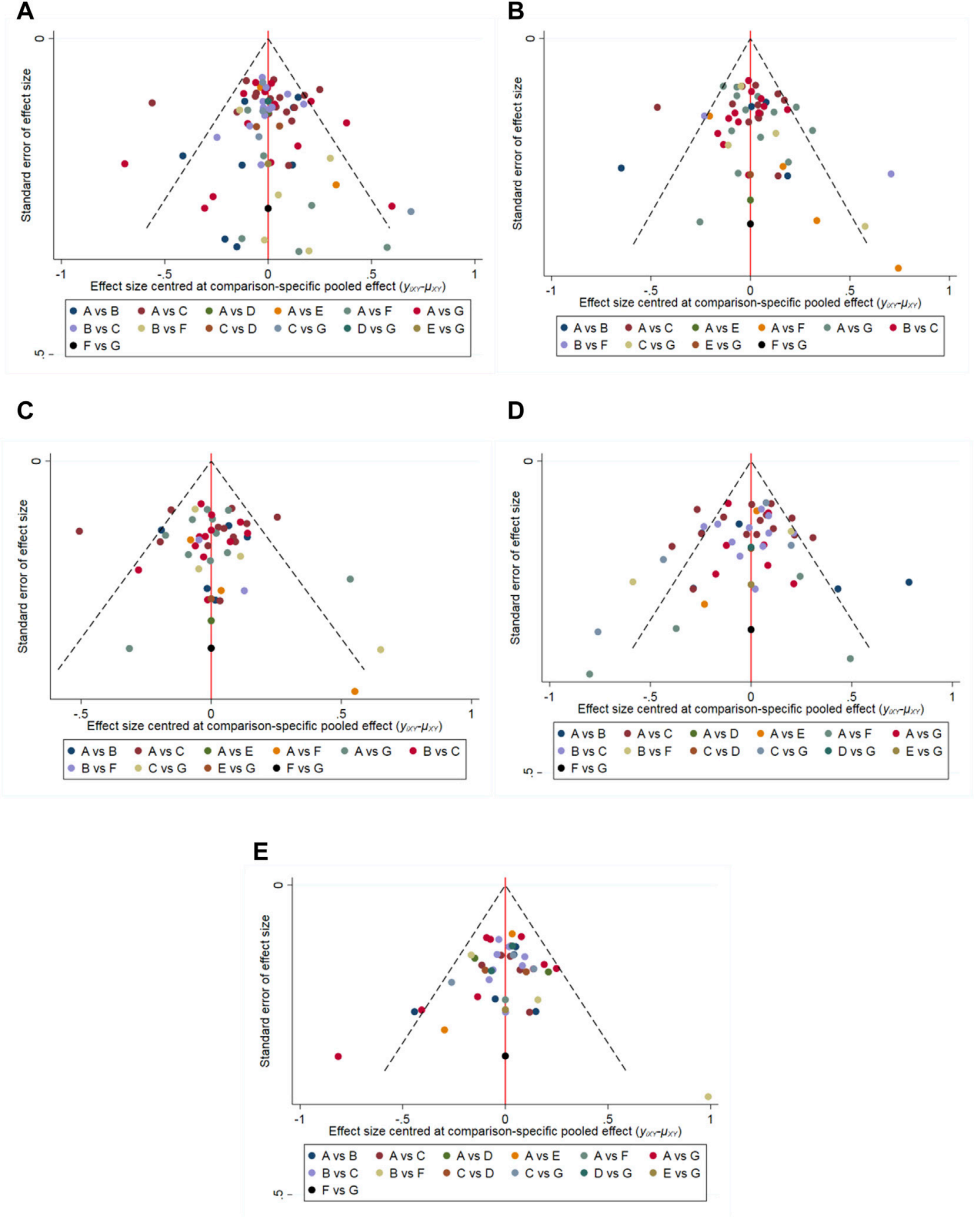
Funnel-plots **(A)** International Prostrate Symptom Score (IPSS) (Egger’s test, *p* = 0.095); **(B)** IPSS-storage (Egger’s test, *p* = 0.067); **(C)** IPSS-voiding (Egger’s test, *p* = 0.103); **(D)** Maximum flow rate (Qmax) (Egger’s test, *p* = 0.104); **(E)** Post-void residual urine (PVR) (Egger’s test, *p* = 0.901). A: α1-adrenoceptor antagonists (ABs); B: phosphodiesterase five inhibitors (PDE5-Is); C: Placebo; D: muscarinic receptor antagonists (MRAs); E: ABs + B3As; F: ABs + PDE5-Is; and G: ABs + MRAs.

The results of the node-splitting analysis showed that for improving the total IPSS, the difference between the direct and indirect comparisons of the placebo and ABs + MRAs was statistically significant (*p* = 0.0402), suggesting that there was inconsistency. *P* was greater than 0.05 in all other comparisons. Funnel plots for each outcome index (Figure 7) showed that most of the studies were evenly distributed in 95% CI, and the Egger’s test showed that *P* was greater than 0.05, indicating that there was no significant publication bias.

## 4 Discussion

The present NMA showed that ABs + PDE5-Is had the greatest advantage for improving the total IPSS score in patients with BPH/LUTS. We further found that the combination therapy was effective in reducing both the IPSS-storage and IPSS-voiding sub-scores. PDE5-Is play a therapeutic role mainly by regulating the function of the NO/cGMP pathway. Nitric oxide (NO) is synthesized from L-arginine by the catalytic action of NO synthase and diffuses into the cell to catalyze cGMP production. cGMP can activate protein kinases, ion channels, and cGMP-phosphodiesterase conjugates; deplete Ca^2+^; reduce sensitivity to contractile proteins; and relax smooth muscles (Held and Dostmann, 2012). PDE5-Is can increase intracellular cGMP concentration and prolong its activity, promote the relaxation of related smooth muscles (bladder, prostate, and urethra), and then make the urination and urine storage functions of the lower urinary tract reach a new equilibrium, relieving the symptoms of BPH/LUTS (Uckert et al., 2006). The reason underlying the higher therapeutic efficacy of ABs and PDE5-Is may be the synergism between the two drugs. Related studies suggest that PDE5-Is can enhance the effectiveness of ABs in inhibiting the neurogenic contraction of the human peripheral prostate and bladder neck. Similarly, ABs can enhance PDE5-I-mediated relaxation by blocking α1-adrenergic receptors and reducing the sympathetic tone of the penis smooth muscle and the prostate/bladder neck (Angulo et al., 2012).

Our NMA suggested that ABs are still the primary treatment to improve Qmax; AB monotherapy and some combination regimens containing ABs could significantly increase Qmax. Similar to other studies, the use of MRAs and PDE5-Is alone could not improve Qmax (Karami et al., 2016), which may be due to their common mode of action by the relaxation of the detrusor muscles. However, there was a significant increase in Qmax after the addition of ABs, indicating an important role in improving Qmax by reducing prostate tension and bladder outlet dynamic obstruction. In addition, we found that compared with that in AB monotherapy, the use of ABs + PDE5-Is showed an effective increase in Qmax, which was consistent with the results of Mauro’s meta-analysis (Gacci et al., 2012a), suggesting ABs + PDE5-Is as the most suitable therapeutic measure to improve Qmax.

The European Association of Urology recommends the use of a combination of MRAs and ABs in patients with moderate to severe LUTS if monotherapy provides insufficient relief of the storage symptoms. Concurrently, in this NMA, we found that ABs + MRAs ranked second in improving the IPSS storage score. However, there was no effect on the SUCRA, and this combination ranked last in improving the IPSS voiding sub-score, indicating a predominant role for ABs + MRAs in alleviating storage symptoms. Inhibition of muscarinic receptors can reduce the contraction of smooth muscle cells and the sensory threshold of the bladder; therefore, MRAs are used to treat storage symptoms such as urgency and frequency. However, this blocking activity may, in turn, inhibit the bladder’s ability to contract, leading to an increase in residual urine (Li et al., 2014). The PVR analysis of this NMA revealed that the use of ABs + MRAs and MRAs alone might aggravate PVR. The meta-analysis performed by Gong et al. (2015) also identified that ABs + MRAs can aggravate PVR compared with Abs alone, but the effect observed in that meta-analysis was smaller than that in the present NMA. In clinical use, physicians often worry that MRAs will increase residual urine and lead to urinary retention. However, in the SATURN (Van Kerrebroeck et al., 2013a), NEPTUNE (Van Kerrebroeck et al., 2013b), and NEPTUNE II trials (Drake et al., 2015), the incidence of urinary retention was very low in the combined treatment group (no more than 1.3%). Therefore, periodic measurement of PVR during the use of antimuscarinics is still recommended to assess the increase in PVR or the incidence of urinary retention.

In this study, the efficacy of B3As was compared with that of different drugs for the first time. ABs + B3As effectively reduced the total IPSS and had the greatest efficacy in improving the IPSS-storage subgroup score, although this combination did not improve Qmax and PVR. β3-adrenergic receptor agonists can increase cAMP levels by activating bladder smooth muscle β3-adrenergic receptor G protein and adenylate cyclase. cAMP acts as a second messenger and activates protein kinase A by changing the state of cell contraction, causing the detrusor to relax, and reducing the smooth muscle tension of the bladder (Allison and Gibson, 2018). Although ABs + B3As were not found to cause an increase in residual urine in this NMA, it is controversial whether this increase is possible. Related studies have different conclusions. Some studies suggest that the stimulation of β3-adrenergic receptors by B3As allows detrusor relaxation during storage but has little effect on bladder contraction amplitude during urination (Leon et al., 2008). A phase 2 urodynamic study on 200 men with LUTS and bladder outlet obstruction showed that doses of up to 100 mg of mirabegron (B3A) do not adversely affect Qmax or detrusor pressure at Qmax (Nitti et al., 2013). Kaplan et al. (2020) also found that the increase in PVR upon combination therapy is not statistically significant. However, the analyses by Su et al. (2020) and Ichihara et al. (2015) identified that the combined use will cause the average volume change of PVR to be significantly higher than that of tamsulosin (AB) alone. Pairwise meta-analysis in this study suggested a similar conclusion. Therefore, more randomized controlled trials are needed to further explore the effect of ABs + B3As on residual urine.

This study focused on the short-term efficacy of the drug. As 5α- reductase inhibitor takes a long time to take effect, it was not included in this NMA. It should be noted, however, that α1-blockers and 5α-reductase inhibitors remain the most recommended drugs for the treatment of BPH in terms of long-term efficacy (Xu et al., 2020). In addition, the volume of the prostate will continue to increase with the age of patients. When the volume of the prostate reaches a certain level, the curative effect of drug therapy will obviously decrease. At this time, surgical treatment will be inevitable. Currently, there are various surgical treatment methods for BPH. According to the results of the latest NMA, in patients with BPH >60 ml, compared with those of open prostatectomy and unipolar transurethral prostatectomy, holmium and diode laser enucleation of the prostate, bipolar enucleation of the prostate, and laparoscopic simple prostatectomy appear to have superior efficacy and safety (Wang et al., 2021).

This study has some limitations. First, only a few single-drug MRA studies were included, which may affect the final analysis results. Second, the node-splitting analysis found incompatibility between the direct and indirect effects of some indicators, such as the comparison between placebo and ABs + MRAs in improving the total IPSS. It is recommended that high-quality RCTs be used to validate our NMA results.

## 5 Conclusion

Overall, the combination therapies showed more positive results. Although ABs + PDE5-Is was the most successful treatment for improving the total IPSS score and the IPSS-voiding sub-score in patients with BPH/LUTS, ABs + B3As was the most effective treatment for improving the IPSS-storage sub-score. ABs are still the basic therapeutic measure to increase Qmax, with improved efficacy upon combination with PDE5-Is. None of the six therapeutic regimens effectively reduced PVR, and MRA monotherapy and a combination of ABs + MRAs may lead to an increase in PVR (Gacci et al., 2012b).

## Data Availability

The original contributions presented in the study are included in the article/Supplementary Material, further inquiries can be directed to the corresponding author.
