# Supplementary material for: Comparative Efficacy of Different Drugs for Lower Urinary Tract Symptoms due to Benign Prostatic Hyperplasia: A Bayesian Network Meta-Analysis
Source: Front Pharmacol. 2022 Mar 7;13:763184. doi: 10.3389/fphar.2022.763184 (PMC8940212; doi:10.3389/fphar.2022.763184)
Supplement: Supplementary file 1 [file DataSheet1.docx]

Supplementary Material

**Supplementary Table S1：Basic characteristics of the included studies**  2

**Supplementary Table S2：Pairwise meta-analysis and subgroup analysis**  9

**Supplementary Figure S1：Evaluation of literature quality** 16

**Supplementary Figure S2：IPSS forest plots with treatment time ≥12W** 17

**Supplementary Figure S3：IPSS forest plots with treatment time <12W** 18

**Supplementary Figure S4：IPSS SUCRA plots with treatment time ≥12W**  19

**Supplementary Figure S5：IPSS SUCRA plots with treatment time ＜12W**  20

**Supplementary Figure S6：Qmax forest plots with treatment time ≥12W**  21

**Supplementary Figure S7：Qmax forest plots with treatment time <12W**  22

**Supplementary Figure S8：Qmax SUCRA plots with treatment time ≥12W** 23

**Supplementary Figure S9：Qmax SUCRA plots with treatment time＜12W**  24

**Supplementary Figure S10：PVR forest plots with treatment time ≥12W**  25

**Supplementary Figure S11：PVR SUCRA plots with treatment time ≥12W**  26

| **Supplementary Table S1：Basic characteristics of the included studies.** | | | | | |
| --- | --- | --- | --- | --- | --- |
| **Study** | **Intervention** | **Follow-up** | **Outcome** | **Country** | **Race** |
| MacDiarmid et al. (2008) | Group A(n=206):tamsulosin(0.4mg/d) | 12w | IPSS,PVR,QOL | USA | White,Black,Asian,Hispanic,Other |
|  | Group B(n=203):tamsulosin(0.4mg/d)+oxybutynin(10mg/d) |  |  |  |  |
| Kaplan et al. (2009) | Group A(n=202):tamsulosin(0.4mg/d) | 12w | IPSS | USA | White,Black,Other |
|  | Group B(n=195):tamsulosin(0.4mg/d)+solifenacin(5mg/d) |  |  |  |  |
| Seo et al. (2011) | Group A(n=29):tamsulosin(0.2mg/d) | 12w | IPSS,QOL | South Korea | - |
|  | Group B(n=27):tamsulosin(0.2mg/d)+solifenacin(5mg/d) |  |  |  |  |
| Yamaguchi et al. (2011) | Group A(n=215):tamsulosin(0.2mg/d) | 12w | IPSS,PVR,QOL,Qmax | Japan | - |
|  | Group B(n=210):tamsulosin(0.2mg/d)+solifenacin(2.5mg/d) |  |  |  |  |
|  | Group C(n=213):tamsulosin(0.2mg/d)+solifenacin(5mg/d) |  |  |  |  |
| Kaplan et al. (2012) | Group A(n=473):α-blocker | 12w | IPSS,QOL | multi-country multi-center | White,Black,Asian,Other |
|  | Group B(n=474):α-blocker+fesoterodine(4-8mg/d) |  |  |  |  |
| Kaplan et al. (2013) | Group A(n=74):placebo | 12w | IPSS,PVR,Qmax | multi-country multi-center | White,Black,Asian,Other |
|  | Group B(n=74):tamsulosin(0.4mg/d)+solifenacin(6mg/d) |  |  |  |  |
|  | Group C(n=74):tamsulosin(0.4mg/d)+solifenacin(9mg/d) |  |  |  |  |
| Van et al. (2013a) | Group A(n=92):placebo | 12w | IPSS,PVR | multi-country multi-center | White,Black,Asian,Other |
|  | Group B(n=179):tamsulosin(0.4mg/d) |  |  |  |  |
|  | Group C(n=43)solifenacin(3mg/d) |  |  |  |  |
|  | Group D(n=44)solifenacin(6mg/d) |  |  |  |  |
|  | Group E(n=44)solifenacin(9mg/d) |  |  |  |  |
|  | Group F(n=180):tamsulosin(0.4mg/d)+solifenacin(3mg/d) |  |  |  |  |
|  | Group G(n=180):tamsulosin(0.4mg/d)+solifenacin(6mg/d) |  |  |  |  |
|  | Group H(n=176):tamsulosin(0.4mg/d)+solifenacin(9mg/d) |  |  |  |  |
| Van et al. (2013b) | Group A(n=341):placebo | 12w | IPSS,QOL,Qmax | multi-country multi-center | White,Black,Asian,Other |
|  | Group B(n=327):tamsulosin(0.4mg/d) |  |  |  |  |
|  | Group C(n=339):tamsulosin(0.4mg/d)+solifenacin(6mg/d) |  |  |  |  |
|  | Group D(n=327):tamsulosin(0.4mg/d)+solifenacin(9mg/d) |  |  |  |  |
| Takeda et al. (2013) | Group A(n=154):tamsulosin(0.2mg/d) | 12w | IPSS,PVR,QOL,Qmax | Japan | - |
|  | Group B(n=154):tamsulosin(0.2mg/d)+imidafenacin(0.2mg/d) |  |  |  |  |
| Lee et al. (2014) | Group A(n=80):tamsulosin(0.2mg/d) | 4w | IPSS,PVR,QOL,Qmax | South Korea | - |
|  | Group B(n=76):tamsulosin(0.2mg/d)+solifenacin(5mg/d) |  |  |  |  |
| Ichihara et al. (2015) | Group A(n=38):tamsulosin(0.2mg/d) | 8w | IPSS,PVR,QOL,Qmax | Japan | - |
|  | Group B(n=38):tamsulosin(0.2mg/d)+ Mirabegron(50mg/d) |  |  |  |  |
| Cho et al. (2017) | Group A(n=111):alfuzosin(10mg/d) | 12w | IPSS,PVR,QOL,Qmax | South Korea | - |
|  | Group B(n=110):alfuzosin(10mg/d)+ imidafenacin(0.2mg/d) |  |  |  |  |
| Matsukawa et al. (2017) | Group A(n=38):silodosin(8mg/d) | 12w | IPSS,PVR,QOL,Qmax | Japan | - |
|  | Group B(n=38):silodosin(0.2mg/d)+propiverine(20mg/d) |  |  |  |  |
| Matsukawa et al. (2019) | Group A(n=60):silodosin(8mg/d)+fesoterodine(4mg/d) | 12w | IPSS,PVR,QOL,Qmax | Japan | - |
|  | Group B(n=60):silodosin(8mg/d)+mirabegron(50mg/d) |  |  |  |  |
| Urakami et al. (2019) | Group A(n=38):α-blocker+tadalafil(5mg/d) | 12w | IPSS,PVR,QOL,Qmax | Japan | - |
|  | Group B(n=37):α-blocker+solifenacin(5mg/d) |  |  |  |  |
| Bechara et al. (2008) | Group A(n=14):tamsulosin(0.4mg/d)+tadalafil(20mg/d) | 12w | IPSS | Argentina | - |
|  | Group B(n=13):tamsulosin(0.4mg/d) |  |  |  |  |
| Dmochowski et al. (2010) | Group A(n=101):placebo | 12w | IPSS,PVR,Qmax | USA，Canada | Black,White,Hispanic,Other |
|  | Group B(n=99):tadalafil(50mg/d) |  |  |  |  |
| Egerdie et al. (2012) | Group A(n=200):placebo | 12w | IPSS,PVR,QOL,Qmax | multi-country multi-center | White,Black,Asian,Other |
|  | Group B(n=198):tadalafil(2.5mg/d) |  |  |  |  |
|  | Group C(n=208):tadalafil(5mg/d) |  |  |  |  |
| Kim et al. (2011) | Group A(n=51):placebo | 12w | IPSS,PVR,QOL,Qmax | South Korea | - |
|  | Group B(n=49):tamsulosin(0.2mg/d) |  |  |  |  |
|  | Group C(n=51):tadalafil(5mg/d) |  |  |  |  |
| McVary et al. (2007a) | Group A(n=143):placebo | 12w | IPSS,PVR,QOL,Qmax | USA | Black,White,Hispanic,Other |
|  | Group B(n=138):tadalafil(5mg/d) |  |  |  |  |
| McVary et al. (2007b) | Group A(n=179):placebo | 12w | IPSS,PVR,QOL,Qmax | USA | Black,White,Asian,Other |
|  | Group B(n=187):sildenafil(50-100mg/d) |  |  |  |  |
| Oelke et al. (2012） | Group A(n=172):placebo | 12w | IPSS,PVR,QOL,Qmax | multi-country multi-center | Black，White，American Indian |
|  | Group B(n=168):tamsulosin(0.4mg/d) |  |  |  |  |
|  | Group C(n=171):tadalafil(5mg/d) |  |  |  |  |
| Porst et al. (2011) | Group A(n=164):placebo | 12w | IPSS,PVR,QOL,Qmax | multi-country multi-center | Black，White，American Indian |
|  | Group B(n=161):tadalafil(5mg/d) |  |  |  |  |
| Roehrborn et al.(2008) | Group A(n=212):placebo | 12w | IPSS,QOL,Qmax | multi-country multi-center | Black，White，Hispanic |
|  | Group B(n=209):tadalafil(2.5mg/d) |  |  |  |  |
|  | Group C(n=212):tadalafil(5mg/d) |  |  |  |  |
|  | Group D(n=216):tadalafil(10mg/d) |  |  |  |  |
|  | Group E(n=209):tadalafil(20mg/d) |  |  |  |  |
| Stief et al. (2008) | Group A(n=113):placebo | 8w | IPSS,PVR,Qmax | Germany | White，Black,Asian |
|  | Group B(n=109):vardenafil(5mg/d) |  |  |  |  |
| Takeda et al. (2012) | Group A(n=140):placebo | 12w | IPSS,QOL,Qmax | Japan | - |
|  | Group B(n=142):tadalafil(2.5mg/d) |  |  |  |  |
|  | Group C(n=140):tadalafil(5mg/d) |  |  |  |  |
| Yokoyama et al. (2013) | Group A(n=154):placebo | 12w | IPSS,PVR,QOL,Qmax | Japan | - |
|  | Group B(n=151):tadalafil(2.5mg/d) |  |  |  |  |
|  | Group C(n=155):tadalafil(5mg/d) |  |  |  |  |
|  | Group D(n=152):tamsulosin(0.2mg/d) |  |  |  |  |
| Tuncel et al. (2010) | Group A(n=20):sildenafil(25mg 4d/w) | 8w | IPSS,QOL | Turkey | - |
|  | Group B(n=20):tamsulosin(0.4mg/d) |  |  |  |  |
|  | Group C(n=20):tamsulosin(0.4mg/d)+sildenafil(25mg 4d/w) |  |  |  |  |
| Gacci et al. (2012) | Group A(n=30):tamsulosin(0.4mg/d) | 12w | IPSS,Qmax | Italy | - |
|  | Group B(n=30):tamsulosin(0.4mg/d)+vardenafil(10mg/d) |  |  |  |  |
| Regadas et al. (2013) | Group A(n=20):tamsulosin(0.4mg/d) | 30d | IPSS,Qmax | Brazil | - |
|  | Group B(n=20):tamsulosin(0.4mg/d)+tadalafil(5mg/d) |  |  |  |  |
| Karami et al. (2016) | Group A(n=61):tamsulosin(0.4mg/d) | 3m | IPSS,PVR,Qmax | Iran | - |
|  | Group B(n=61):tadalafil(20mg/d) |  |  |  |  |
|  | Group C(n=61):tamsulosin(0.4mg/d)+tadalafil(20mg/d) |  |  |  |  |
| Kim et al. (2017) | Group A(n=171):tadalafil(5mg/d) | 12w | IPSS,PVR,QOL,Qmax | South Korea | - |
|  | Group B(n=170):tamsulosin(0.2mg/d)+tadalafil(5mg/d) |  |  |  |  |
|  | Group C(n=169):tamsulosin(0.4mg/d)+tadalafil(5mg/d) |  |  |  |  |
| Takeda et al. (2017) | Group A(n=86):α-blocker | 8w | IPSS,QOL | Japan | - |
|  | Group B(n=85):α-blocker+tadalafil(5mg/d) |  |  |  |  |
| Brock et al. (2013) | Group A(n=545):placebo | 12w | IPSS,QOL | multi-country multi-center | - |
|  | Group B(n=544):tadalafil(5mg/d) |  |  |  |  |
| Pogula et al. (2019) | Group A(n=50):tamsulosin(0.4mg/d) | 12w | IPSS,PVR,QOL,Qmax | India | - |
|  | Group B(n=50):tadalafil(5mg/d) |  |  |  |  |
| Zhang et al. (2019) | Group A(n=361):placebo(0.4mg/d) | 12w | IPSS | China,South Korea | - |
|  | Group B(n=185):tamsulosin(0.2mg/d) |  |  |  |  |
|  | Group C(n=363):tadalafil(5mg/d) |  |  |  |  |
| Kaplan et al (2020) | Group A(n=354):tamsulosin(0.4mg/d) | 12w | IPSS,PVR,Qmax | multi-country multi-center | White，Black,Asian |
|  | Group B(n=352):tamsulosin+mirabegron(25-50mg/d) |  |  |  |  |
| Roehrborn et al. (2009) | Group A(n=215):placebo(0.4mg/d) | 12w | IPSS,PVR,Qmax | USA | White，Black,Other |
|  | Group B(n=209):tamsulosin(0.4mg/d) |  |  |  |  |
|  | Group C(n=210):tolterodine(4mg/d) |  |  |  |  |
|  | Group D(n=217):tamsulosin(0.4mg/d)+tolterodine(4mg/d) |  |  |  |  |
| Van et al. (2000) | Group A(n=154):placebo | 3m | IPSS,QOL,Qmax | multi-country multi-center | - |
|  | Group B(n=143):alfuzosin(10mg/d) |  |  |  |  |
|  | Group C(n=150):alfuzosin(7.5mg/d) |  |  |  |  |
| Roehrborn (2001) | Group A(n=175):placebo | 3m | IPSS,QOL,Qmax | USA，Canada | - |
|  | Group B(n=176):alfuzosin(10mg/d) |  |  |  |  |
|  | Group C(n=177):alfuzosin(15mg/d) |  |  |  |  |
| Roehrborn et al. (2003) | Group A(n=482):placebo | 12w | IPSS,QOL,Qmax | multi-country multi-center | - |
|  | Group B(n=473):alfuzosin(10mg/d) |  |  |  |  |
| Andersen et al. (2000) | Group A(n=151):placebo | 13w | IPSS,QOL,Qmax | multi-country multi-center | White,Asian,Other |
|  | Group B(n=311):doxazosin(4-8mg/d) |  |  |  |  |
|  | Group C(n=310):doxazosin GITS(1-8mg/d) |  |  |  |  |
| Kirby et al. (2001) | Group A(n=155):placebo | 13w | IPSS,Qmax | multi-country multi-center | White,Black,Asian,Other |
|  | Group B(n=640):doxazosin(4-8mg/d) |  |  |  |  |
|  | Group C(n=651):doxazosin GITS(1-8mg/d) |  |  |  |  |
| Chapple et al. (2009) | Group A(n=323):a-blocker | 12w | IPSS,QOL,PVR,Qmax | multi-country multi-center | White,Black,Asian,Other |
|  | Group B(n=329):a-blocker+tolterodine(4mg/d) |  |  |  |  |
| Singh et al. (2015) | Group A(n=30):tamsulosin(0.4mg/d) | 8w | IPSS,PVR | India | - |
|  | Group B(n=30):tamsulosin(0.4mg/d)+darifenacin(7.5mg/d) |  |  |  |  |
| Chapple et al. (2011) | Group A(n=190):placebo | 12w | IPSS,Qmax | multi-country multi-center | - |
|  | Group B(n=381):silodosin(8mg/d) |  |  |  |  |
|  | Group C(n=384):tamsulosin(0.4mg/d) |  |  |  |  |
| Kawabe et al. (2006) | Group A(n=89):placebo | 12w | IPSS,QOL,Qmax | Japan | - |
|  | Group B(n=175):silodosin(4mg bid/d) |  |  |  |  |
|  | Group C(n=192):tamsulosin(0.2mg/d) |  |  |  |  |
| Chung et al. (2018) | Group A(n=167):placebo | 12w | IPSS,QOL | South Korea | - |
|  | Group B(n=162):tamsulosin(0.4mg/d) |  |  |  |  |
|  | Group C(n=162):tamsulosin(0.2mg/d) |  |  |  |  |
| Roehrborn et al. (2011) | Group A(n=457):placebo | 12w | IPSS,QOL,Qmax | USA | - |
|  | Group B(n=466):silodosin(8mg/d) |  |  |  |  |
| Resnick et al. (2007) | Group A(n=186):placebo | 4w | IPSS,QOL,Qmax | USA，UK | - |
|  | Group B(n=186):alfuzosin(10mg/d) |  |  |  |  |
| Nordling 2005 | Group A(n=154):placebo | 12w | IPSS,Qmax | multi-country multi-center | - |
|  | Group B(n=154):alfuzosin(10mg/d) |  |  |  |  |
|  | Group C(n=159):alfuzosin(15mg/d) |  |  |  |  |
|  | Group D(n=158):tamsulosin(0.4mg/d) |  |  |  |  |
| Cai et al. (2016) | Group A(n=38):placebo | 12w | IPSS,Qmax | China | - |
|  | Group B(n=38):tamsulosin(0.2mg/d) |  |  |  |  |
|  | Group C(n=38):tolterodine(4mg/d) |  |  |  |  |
|  | Group D(n=38):tamsulosin(0.2mg/d)+tolterodine(4mg/d) |  |  |  |  |
| Maruyama et al. (2006) | Group A(n=53):naftopidil(25-75mg/d) | 12w | IPSS | Japan | - |
|  | Group B(n=48):naftopidil(25-75mg/d)+anticholinergic agent |  |  |  |  |
| Lee et al. (2017) | Group A(n=44):tamsulosin(0.2mg/d) | 12w | IPSS | South Korea | - |
|  | Group B(n=55):tamsulosin(0.2mg/d)+solifenacin(5mg/d) |  |  |  |  |
|  | Group C(n=47):tamsulosin(0.2mg/d)+solifenacin(10mg/d) |  |  |  |  |
| Kaplan et al. (2007) | Group A(n=20):alfuzosin(10mg/d) | 12w | IPSS | USA | - |
|  | Group B(n=21):sildenafil(25mg/d) |  |  |  |  |
|  | Group C(n=21):alfuzosin(10mg/d)+sildenafil(25mg/d) |  |  |  |  |

| **Supplementary Table S2：Pairwise meta-analysis and subgroup analysis**  Bold numbers represent statistically significant. include α1-adrenoceptor antagonists (ABs), muscarinic receptor antagonists (MRAs), phosphodiesterase 5 inhibitors (PDE5-Is), and β3-adrenoceptor agonists (B3As). IPSS:International Prostate Symptom Score, Qmax:maximum flow rate, PVR:post-void residual urine. | | | | | | | | | | | | |
| --- | --- | --- | --- | --- | --- | --- | --- | --- | --- | --- | --- | --- |
| **COMPARISON** | | | **IPSS** | | | | **Qmax** | | | **PVR** | | |
|  |  |  | **N of RCTs** | | **IPSS** | | **N of RCTs** | **Qmax** | | **N of RCTs** | **PVR** | |
| **ABs+MRAs VS ABs** | | | 15 | | -0.12 [-0.27, 0.02] | | 8 | -0.02 [-0.10, 0.05] | | 11 | **0.75 [0.33, 1.16]** | |
| subgroup | tamsulosin+oxybutynin VS tamsulosin | | 1 | | **-0.27 [-0.46, -0.07]** | | - | - | | 1 | **0.23 [0.04, 0.42]** | |
|  | tamsulosin+solifenacin VS tamsulosin | | 7 | | -0.10 [-0.38, 0.18] | | 3 | -0.00 [-0.10, 0.10] | | 3 | **4.92 [3.19, 6.66]** | |
|  | tamsulosin+imidafenacin VS tamsulosin | | 1 | | **-0.44 [-0.70, -0.18]** | | 1 | 0.15 [-0.21, 0.50] | | 1 | 0.07 [-0.18, 0.32] | |
|  | silodosin+propiverine VS silodosin | | 1 | | -0.07 [-0.46, 0.31] | | 1 | -0.24 [-0.62, 0.15] | | 1 | **0.66 [0.26, 1.05]** | |
|  | tamsulosin+darifenacin VS tamsulosin | | 1 | | **-0.65 [-1.17, -0.13]** | | - | - | | 1 | **1.06 [0.51, 1.60]** | |
|  | alfuzosin+imidafenacin VS alfuzosin | | 1 | | 0.04 [-0.23, 0.30] | | 1 | -0.09 [-0.35, 0.18] | | 1 | 0.01 [-0.26, 0.27] | |
|  | tamsulosin+tolterodine VS tamsulosin | | - | | - | | 1 | 0.10 [-0.17, 0.36] | | 1 | 0.12 [-0.15, 0.38] | |
|  | tamsulosin+propiverine VS tamsulosin | | - | | - | | - | - | | 1 | **0.39 [0.04, 0.74]** | |
| **ABs+MRAs VS Placebo** | | | 5 | | **-0.24 [-0.34, -0.15]** | | 4 | 0.31 [-0.03, 0.65] | | 3 | **0.19 [0.04, 0.34]** | |
| subgroup | tamsulosin+solifenacin VS placebo | | 3 | | **-0.18 [-0.29, -0.08]** | | 2 | 0.30 [-0.19, 0.80] | | 2 | **0.26 [0.08, 0.44]** | |
|  | tamsulosin+tolterodine VS placebo | | 2 | | **-0.54 [-0.78, -0.30]** | | 2 | 0.39 [-0.54, 1.31] | | 1 | 0.06 [-0.21, 0.32] | |
| **ABs VS Placebo** | | | 16 | | **-0.35 [-0.44, -0.26]** | | 15 | **0.24 [0.15, 0.33]** | | 5 | **-0.13 [-0.25, -0.02]** | |
| subgroup | tamsulosin VS placebo | | 9 | | **-0.34 [-0.52, -0.16]** | | 8 | 0.13 [-0.03, 0.29] | | 5 | **-0.13 [-0.25, -0.02]** | |
|  | alfuzosin VS placebo | | 5 | | **-0.32 [-0.40, -0.24]** | | 5 | **0.34 [0.26, 0.43]** | | **-** | - | |
|  | doxazosin VS placebo | | 2 | | **-0.42 [-0.54, -0.30]** | | 2 | **0.38 [0.26, 0.50]** | | **-** | - | |
|  | silodosin VS placebo | | 2 | | **-0.46 [-0.59, -0.34]** | | 2 | **0.30 [0.18, 0.42]** | | **-** | - | |
| **MRAs VS Placebo** | | | 1 | | -0.04 [-0.32, 0.23] | | 1 | -0.03 [-0.31, 0.24] | | 1 | **0.30 [0.03, 0.57]** | |
| subgroup | solifenacin VS placebo | | 1 | | -0.04 [-0.32, 0.23] | | 1 | -0.03 [-0.31, 0.24] | | 1 | **0.30 [0.03, 0.57]** | |
| **MRAs VS ABs** | | | 1 | | 0.19 [-0.04, 0.42] | | 1 | 0.12 [-0.15, 0.40] | | 1 | **0.53 [0.30, 0.76]** | |
| subgroup | solifenacin VS tamsulosin | | 1 | | 0.19 [-0.04, 0.42] | | 1 | 0.12 [-0.15, 0.40] | | 1 | **0.53 [0.30, 0.76]** | |
| **ABs+MRAs VS MRAs** | | | 1 | | -0.14 [-0.33, 0.06] | | 1 | -0.03 [-0.30, 0.25] | | 1 | -0.15 [-0.34, 0.04] | |
| subgroup | tamsulosin+solifenacin VS solifenacin | | 1 | | -0.14 [-0.33, 0.06] | | 1 | -0.03 [-0.30, 0.25] | | 1 | -0.15 [-0.34, 0.04] | |
| **ABs+B3As VS ABs** | | | 2 | | -0.05 [-0.20, 0.09] | | 2 | -0.10 [-0.25, 0.05] | | 2 | **0.25 [0.10, 0.39]** | |
| subgroup | tamsulosin+mirabegron VS tamsulosin | | 2 | | -0.05 [-0.20, 0.09] | | 2 | -0.10 [-0.25, 0.05] | | 2 | **0.25 [0.10, 0.39]** | |
| **ABs+MRAs VS ABs+B3As** | | | 1 | | -0.07 [-0.46, 0.31] | | 1 | -0.09 [-0.48, 0.29] | | 1 | **0.49 [0.09, 0.88]** | |
| subgroup | tamsulosin+solifenacin VS tamsulosin+mirabegron | | 1 | | -0.07 [-0.46, 0.31] | | 1 | -0.09 [-0.48, 0.29] | | 1 | **0.49 [0.09, 0.88]** | |
| **ABs+PDE5-Is VS ABs+MRAs** | | | 1 | | -0.05 [-0.58, 0.47] | | 1 | 0.28 [-0.25, 0.81] | | 1 | **-0.64 [-1.18, -0.10]** | |
| subgroup | α-blocker+tadalafil VS α-blocker+solifenacin | | 1 | | -0.05 [-0.58, 0.47] | | 1 | 0.28 [-0.25, 0.81] | | 1 | **-0.64 [-1.18, -0.10]** | |
| **ABs+PDE5-Is VS ABs** | | | 7 | | **-0.27 [-0.43, -0.12]** | | 4 | 0.40 [-0.11, 0.91] | | 1 | -0.11 [-0.47, 0.25] | |
| subgroup | tamsulosin+tadalafil VS tamsulosin | | 3 | | **-0.40 [-0.69, -0.11]** | | 2 | 0.02 [-0.29, 0.33] | | 1 | -0.11 [-0.47, 0.25] | |
|  | tamsulosin+sildenafil VS tamsulosin | | 1 | | -0.13 [-0.75, 0.49] | | 1 | **1.11 [0.44, 1.78]** | | **-** | - | |
|  | tamsulosin+vardenafil VS tamsulosin | | 1 | | -0.50 [-1.02, 0.02] | | 1 | **0.69 [0.16, 1.22]** | | **-** | - | |
|  | alfuzosin+sildenafil VS alfuzosin | | 1 | | -0.40 [-1.06, 0.26] | |  |  | | - | - | |
| **PDE5-Is VS Placebo** | | | 12 | | **-0.34 [-0.40, -0.29]** | | 11 | 0.02 [-0.04, 0.09] | | 8 | -0.03 [-0.11, 0.06] | |
| subgroup | tadalafil VS placebo | | 10 | | **-0.34 [-0.40, -0.28]** | | 9 | 0.02 [-0.05, 0.09] | | 7 | -0.02 [-0.11, 0.07] | |
|  | sildenafil VS placebo | | 1 | | **-0.36 [-0.58, -0.15]** | | 1 | 0.01 [-0.20, 0.22] | |  |  | |
|  | vardenafil VS placebo | | 1 | | **-0.43 [-0.70, -0.16]** | | 1 | 0.08 [-0.19, 0.35] | | 1 | -0.08 [-0.35, 0.18] | |
| **PDE5-Is VS ABs** | | | 8 | | 0.03 [-0.07, 0.13] | | 5 | -0.31 [-0.66, 0.04] | | 5 | **0.15 [0.02, 0.27]** | |
| subgroup | tadalafil VS tamsulosin | | 6 | | 0.02 [-0.08, 0.12] | | 5 | -0.31 [-0.66, 0.04] | | 5 | **0.15 [0.02, 0.27]** | |
|  | sildenafil VS tamsulosin | | 1 | | 0.23 [-0.39, 0.86] | | - | - | | - | - | |
|  | sildenafil VS alfuzosin | | 1 | | 0.18 [-0.47, 0.82] | | - | - | | - | - | |
| **ABs+PDE5-Is VS PDE5-Is** | | | 4 | | **-0.37 [-0.55, -0.19]** | | 2 | 0.51 [-0.26, 1.27] | | 3 | -0.37 [-0.88, 0.15] | |
| subgroup | tamsulosin+sildenafil VS sildenafil | | 1 | | -0.35 [-0.98, 0.27] | | - | - | | - | - | |
|  | tamsulosin+tadalafil VS tadalafil | | 2 | | **-0.43 [-0.85, -0.01]** | | 2 | 0.51 [-0.26, 1.27] | | 3 | -0.37 [-0.88, 0.15] | |
|  | alfuzosin+sildenafil VS sildenafil | | 1 | | -0.56 [-1.22, 0.09] | | - | - | | - | - | |
| **COMPARISON** | | | | **IPSS-storage** | | | | | **IPSS-voiding** | | |  |
|  |  |  |  | **N of RCTs** | | **IPSS-storage** | | | **N of RCTs** | **IPSS-voiding** | |  |
| **ABs+MRAs VS ABs** | | | | 14 | | **-0.46 [-0.70, -0.23]** | | | 11 | 0.03 [-0.03, 0.09] | |  |
| subgroup | | tamsulosin+oxybutynin VS tamsulosin | | 1 | | **-0.44 [-0.64, -0.24]** | | | - | - | |  |
|  |  | tamsulosin+solifenacin VS tamsulosin | | 6 | | **-0.86 [-1.43, -0.29]** | | | 6 | 0.02 [-0.14, 0.18] | |  |
|  |  | tamsulosin+imidafenacin VS tamsulosin | | 1 | | **-0.52 [-0.78, -0.26]** | | | 1 | -0.03 [-0.29, 0.23] | |  |
|  |  | silodosin+propiverine VS silodosin | | 1 | | -0.15 [-0.53, 0.24] | | | - | - | |  |
|  |  | tamsulosin+darifenacin VS tamsulosin | | - | | - | | | - | - | |  |
|  |  | alfuzosin+imidafenacin VS alfuzosin | | 1 | | -0.12 [-0.38, 0.15] | | | 1 | 0.12 [-0.14, 0.39] | |  |
|  |  | tamsulosin+tolterodine VS tamsulosin | | - | | - | | | - | - | |  |
|  |  | tamsulosin+propiverine VS tamsulosin | | - | | - | | | - | - | |  |
| **ABs+MRAs VS Placebo** | | | | 4 | | **-0.33 [-0.44, -0.22]** | | | 4 | **-0.16 [-0.27, -0.05]** | |  |
| subgroup | | tamsulosin+solifenacin VS placebo | | 2 | | **-0.27 [-0.39, -0.14]** | | | 2 | -0.10 [-0.22, 0.03] | |  |
|  |  | tamsulosin+tolterodine VS placebo | | 2 | | **-0.54 [-0.78, -0.30]** | | | 2 | **-0.38 [-0.62, -0.14]** | |  |
| **ABs VS Placebo** | | | | 12 | | **-0.28 [-0.37, -0.19]** | | | 12 | **-0.32 [-0.44, -0.21]** | |  |
| subgroup | | tamsulosin VS placebo | | 8 | | **-0.27 [-0.42, -0.11]** | | | 8 | **-0.32 [-0.51, -0.14]** | |  |
|  |  | alfuzosin VS placebo | | 4 | | **-0.27 [-0.36, -0.19]** | | | 4 | **-0.25 [-0.34, -0.16]** | |  |
|  |  | doxazosin VS placebo | | - | | - | | | - | - | |  |
|  |  | silodosin VS placebo | | 2 | | **-0.33 [-0.45, -0.21]** | | | 2 | **-0.46 [-0.58, -0.34]** | |  |
| **MRAs VS Placebo** | | | | - | | - | | | - | - | |  |
| subgroup | | solifenacin VS placebo | | - | |  | | |  |  | |  |
| **MRAs VS ABs** | | | | - | | - | | | - | - | |  |
| subgroup | | solifenacin VS tamsulosin | | - | | - | | | - | - | |  |
| **ABs+MRAs VS MRAs** | | | | - | | - | | | - | - | |  |
| subgroup | | tamsulosin+solifenacin VS solifenacin | | - | | - | | | - | - | |  |
| **ABs+B3As VS ABs** | | | | 1 | | **-0.64 [-1.10, -0.18]** | | | 1 | -0.10 [-0.55, 0.35] | |  |
| subgroup | | tamsulosin+mirabegron VS tamsulosin | | 1 | | **-0.64 [-1.10, -0.18]** | | | 1 | -0.10 [-0.55, 0.35] | |  |
| **ABs+MRAs VS ABs+B3As** | | | | 1 | | -0.17 [-0.56, 0.22] | | | 1 | 0.04 [-0.35, 0.42] | |  |
| subgroup | | tamsulosin+solifenacin VS tamsulosin+mirabegron | | 1 | | -0.17 [-0.56, 0.22] | | | 1 | 0.04 [-0.35, 0.42] | |  |
| **ABs+PDE5-Is VS ABs+MRAs** | | | | 1 | | 0.29 [-0.24, 0.82] | | | 1 | -0.10 [-0.63, 0.43] | |  |
| subgroup | | α-blocker+tadalafil VS α-blocker+solifenacin | | 1 | | 0.29 [-0.24, 0.82] | | | 1 | -0.10 [-0.63, 0.43] | |  |
| **ABs+PDE5-Is VS ABs** | | | | 4 | | **-0.39 [-0.76, -0.02]** | | | 3 | **-0.31 [-0.49, -0.12]** | |  |
| subgroup | | tamsulosin+tadalafil VS tamsulosin | | 2 | | **-0.51 [-0.83, -0.19]** | | | 2 | **-0.46 [-0.78, -0.14]** | |  |
|  |  | tamsulosin+sildenafil VS tamsulosin | | - | | - | | | - | - | |  |
|  |  | tamsulosin+vardenafil VS tamsulosin | | 1 | | **-0.54 [-1.06, -0.02]** | | | - | - | |  |
|  |  | alfuzosin+sildenafil VS alfuzosin | | - | | - | | | - | - | |  |
| **PDE5-Is VS Placebo** | | | | 12 | | **-0.27 [-0.33, -0.21]** | | | 12 | **-0.34 [-0.39, -0.28]** | |  |
| subgroup | | tadalafil VS placebo | | 10 | | **-0.26 [-0.32, -0.20]** | | | 10 | **-0.33 [-0.39, -0.27]** | |  |
|  |  | sildenafil VS placebo | | 1 | | **-0.35 [-0.56, -0.13]** | | | 1 | **-0.36 [-0.57, -0.15]** | |  |
|  |  | vardenafil VS placebo | | 1 | | **-0.43 [-0.70, -0.16]** | | | 1 | **-0.36 [-0.63, -0.09]** | |  |
| **PDE5-Is VS ABs** | | | | 5 | | 0.09 [-0.12, 0.29] | | | 5 | -0.02 [-0.12, 0.09] | |  |
| subgroup | | tadalafil VS tamsulosin | | 5 | | 0.09 [-0.12, 0.29] | | | 5 | -0.02 [-0.12, 0.09] | |  |
|  |  | sildenafil VS tamsulosin | | - | | - | | | - | - | |  |
|  |  | sildenafil VS alfuzosin | | - | | - | | | - | - | |  |
| **ABs+PDE5-Is VS PDE5-Is** | | | | 2 | | -0.60 [-1.51, 0.31] | | | 2 | **-0.33 [-0.52, -0.14]** | |  |
| subgroup | | tamsulosin+sildenafil VS sildenafil | | - | | - | | | - | - | |  |
|  |  | tamsulosin+tadalafil VS tadalafil | | 2 | | -0.60 [-1.51, 0.31] | | | 2 | **-0.33 [-0.52, -0.14]** | |  |
|  |  | alfuzosin+sildenafil VS sildenafil | | - | | - | | | - | - | |  |

**
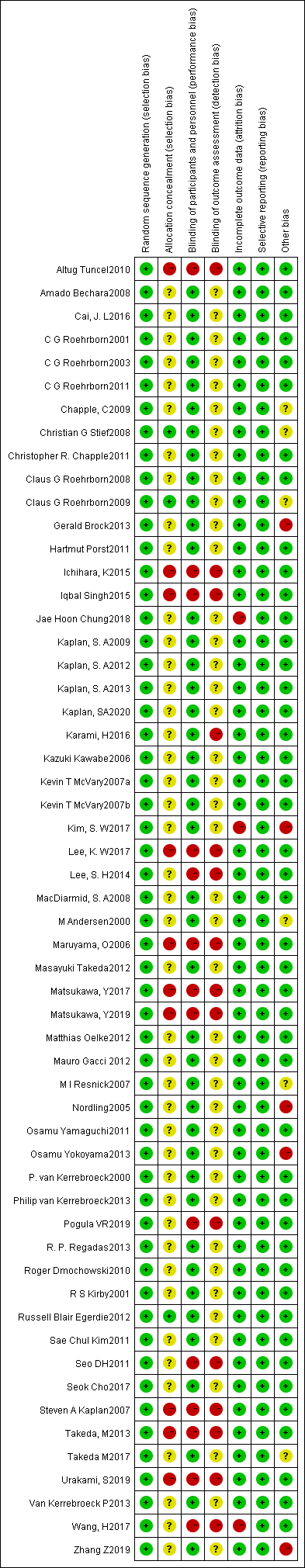
 Supplementary Figure S1：Evaluation of literature quality**

**Supplementary Figure S2：International Prostate Symptom Score (IPSS) forest plots with treatment time ≥12W**

include α1-adrenoceptor antagonists (ABs), muscarinic receptor antagonists (MRAs), phosphodiesterase 5 inhibitors (PDE5-Is), and β3-adrenoceptor agonists (B3As).


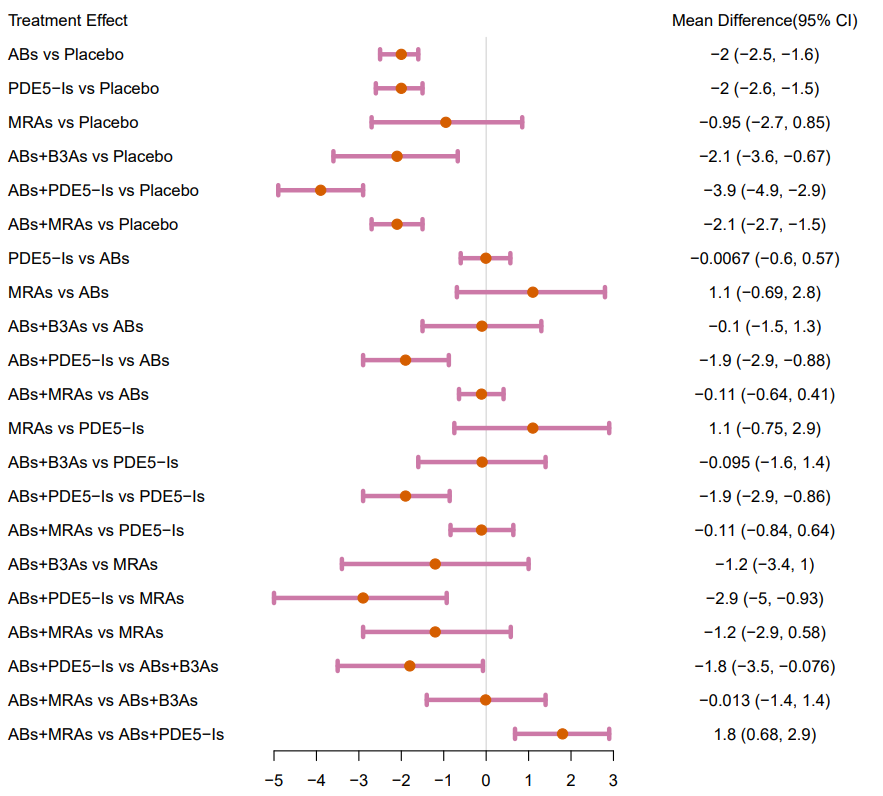


**Supplementary Figure S3：International Prostate Symptom Score (IPSS) forest plots with treatment time <12W**

include α1-adrenoceptor antagonists (ABs), muscarinic receptor antagonists (MRAs), phosphodiesterase 5 inhibitors (PDE5-Is), and β3-adrenoceptor agonists (B3As).


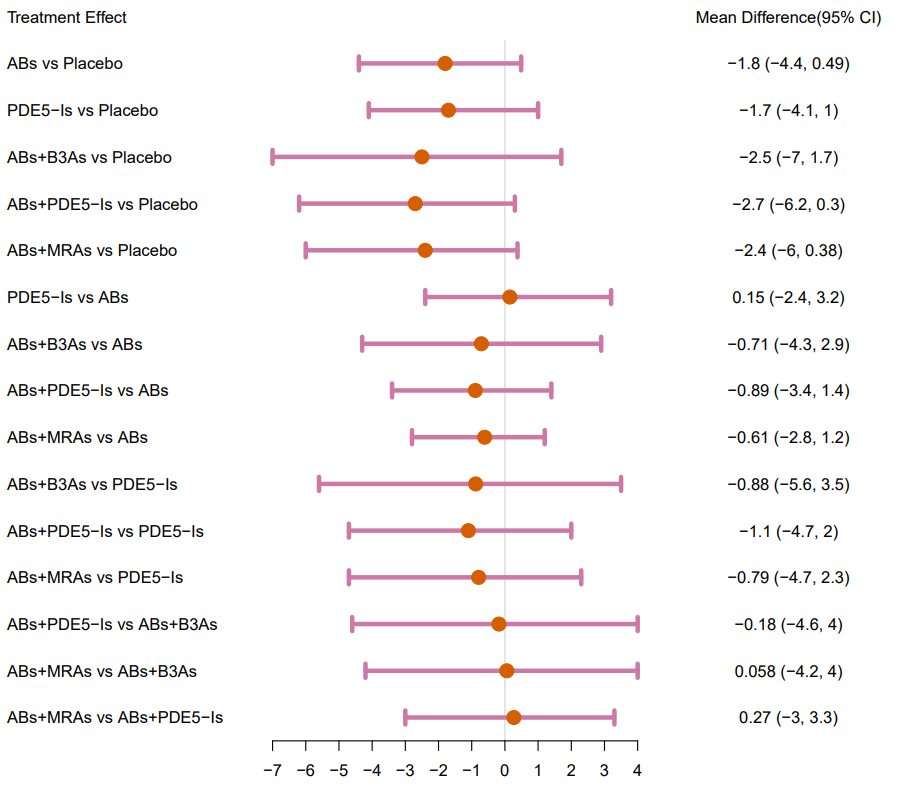


**Supplementary Figure S4：International Prostate Symptom Score (IPSS) Surface under the cumulative ranking curve (SUCRA) plots with treatment time ≥12W**

SUCRA values: α1-adrenoceptor antagonists (ABs) +phosphodiesterase 5 inhibitors (PDE5−Is)（99.6%），ABs+ β3-adrenoceptor agonists (B3As)（61.65%），ABs+muscarinic receptor antagonists (MRAs)（57.91%），PDE5−Is（53.7%），ABs（52.46%），MRAs（22.22%）

**
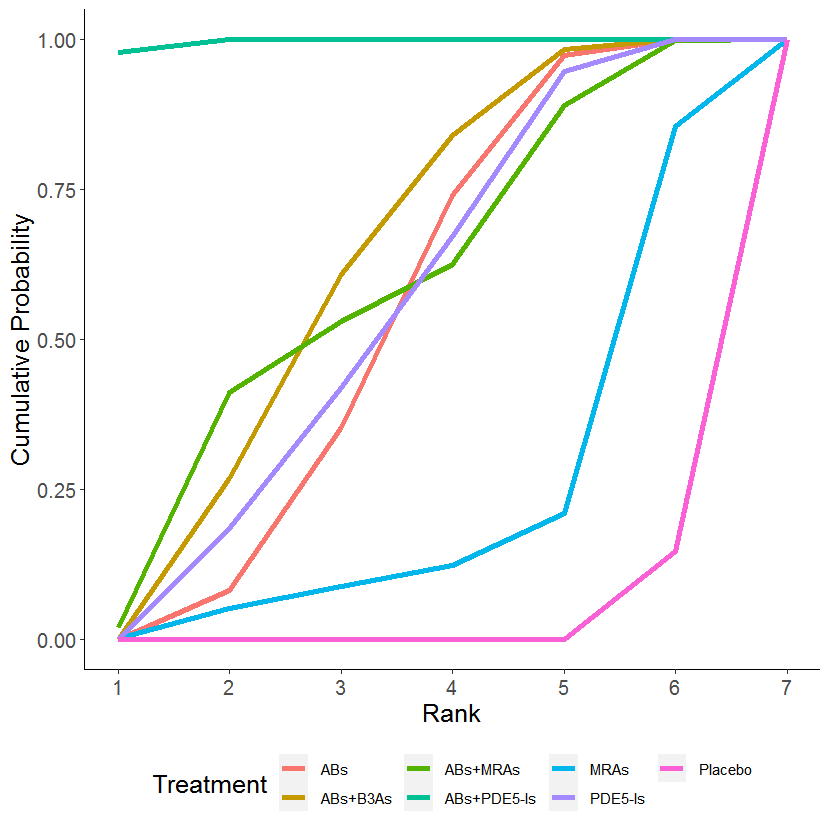
**

**Supplementary Figure S5：International Prostate Symptom Score (IPSS) Surface under the cumulative ranking curve (SUCRA) plots with treatment time ＜12W**

SUCRA values: α1-adrenoceptor antagonists (ABs) +phosphodiesterase 5 inhibitors (PDE5−Is)（73.31%），ABs+ β3-adrenoceptor agonists (B3As)（67.6%），ABs+muscarinic receptor antagonists (MRAs)（64.03%），PDE5−Is（44.66%），ABs（44.12%）


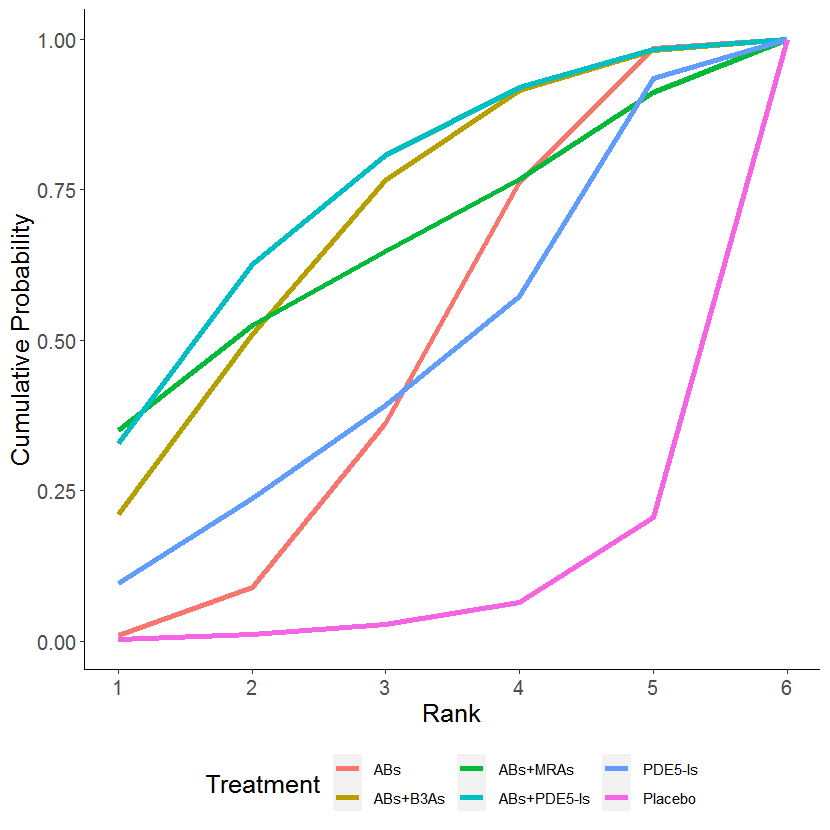


**Supplementary Figure S6：Maximum flow rate (Qmax) forest plots with treatment time ≥12W**

include α1-adrenoceptor antagonists (ABs), muscarinic receptor antagonists (MRAs), phosphodiesterase 5 inhibitors (PDE5-Is), and β3-adrenoceptor agonists (B3As).


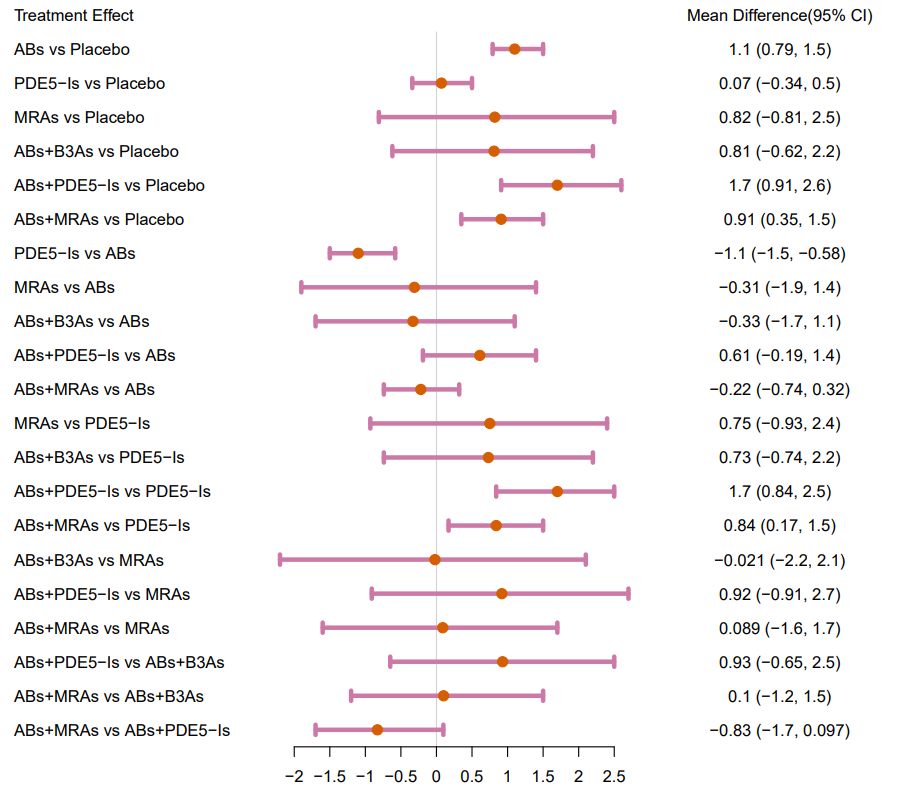


**Supplementary Figure S7：Maximum flow rate (Qmax) forest plots with treatment time <12W**

include α1-adrenoceptor antagonists (ABs), muscarinic receptor antagonists (MRAs), phosphodiesterase 5 inhibitors (PDE5-Is), and β3-adrenoceptor agonists (B3As).


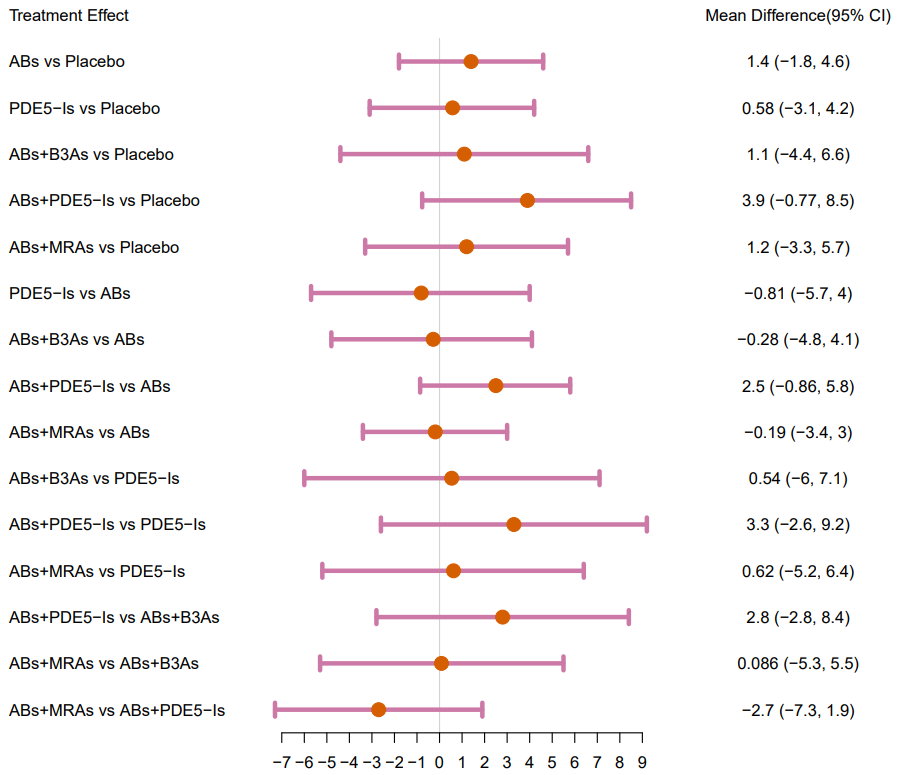


**Supplementary Figure S8：Maximum flow rate (Qmax) surface under the cumulative ranking curve (SUCRA) plots with treatment time ≥12W**

SUCRA values:α1-adrenoceptor antagonists (ABs)+ phosphodiesterase 5 inhibitors (PDE5−Is)（93.53%），ABs（69.83%），ABs+muscarinic receptor antagonists (MRAs)（55.56%），ABs+β3-adrenoceptor agonists (B3As)（52.37%），MRAs（52.23%），PDE5−Is（16.5%）

**
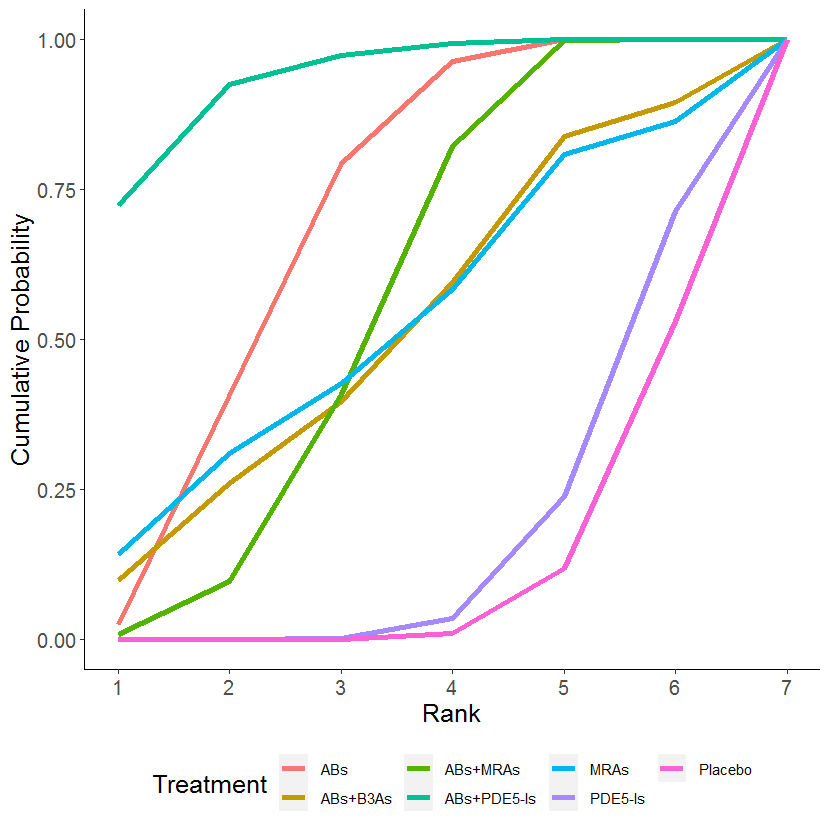
**

**Supplementary Figure S9：Maximum flow rate (Qmax) surface under the cumulative ranking curve (SUCRA) plots with treatment time ＜12W**

SUCRA values:α1-adrenoceptor antagonists (ABs)+ phosphodiesterase 5 inhibitors (PDE5−Is)（91.31%），ABs（54.37%），ABs+muscarinic receptor antagonists (MRAs)（48.25%），ABs+β3-adrenoceptor agonists (B3As)（46.55%），PDE5−Is（37.96%）


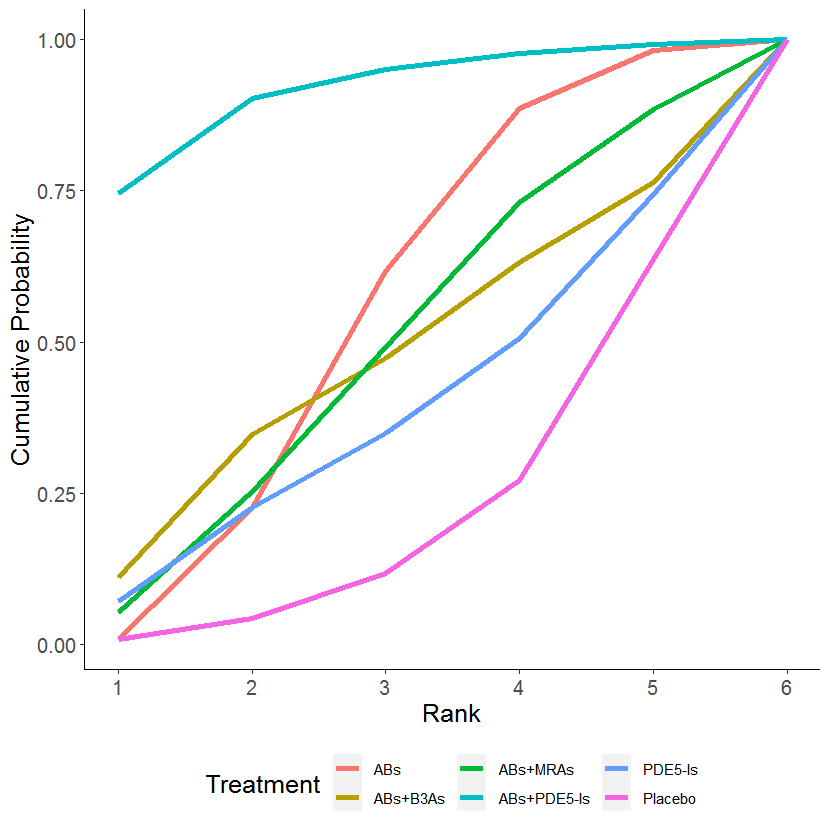


**Supplementary Figure S10：Post-void residual urine (PVR) forest plots with treatment time ≥12W**

include α1-adrenoceptor antagonists (ABs), muscarinic receptor antagonists (MRAs), phosphodiesterase 5 inhibitors (PDE5-Is), and β3-adrenoceptor agonists (B3As).


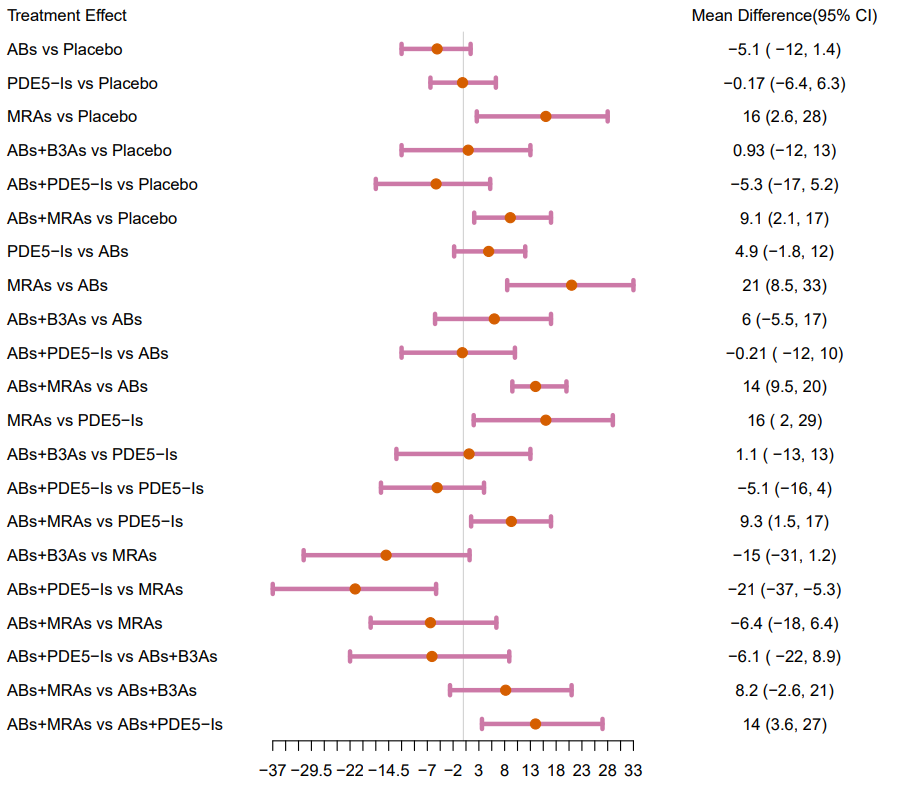


**Supplementary Figure S11：Post-void residual urine (PVR) surface under the cumulative ranking curve (SUCRA) plots with treatment time ≥12W**

SUCRA values:α1-adrenoceptor antagonists (ABs)+ phosphodiesterase 5 inhibitors (PDE5−Is)（86.92%），ABs（83.38%），PDE5−Is（54.56%），ABs+β3-adrenoceptor agonists (B3As)（51.86%），ABs+muscarinic receptor antagonists (MRAs)（15.55%），MRAs（3.66%）

**
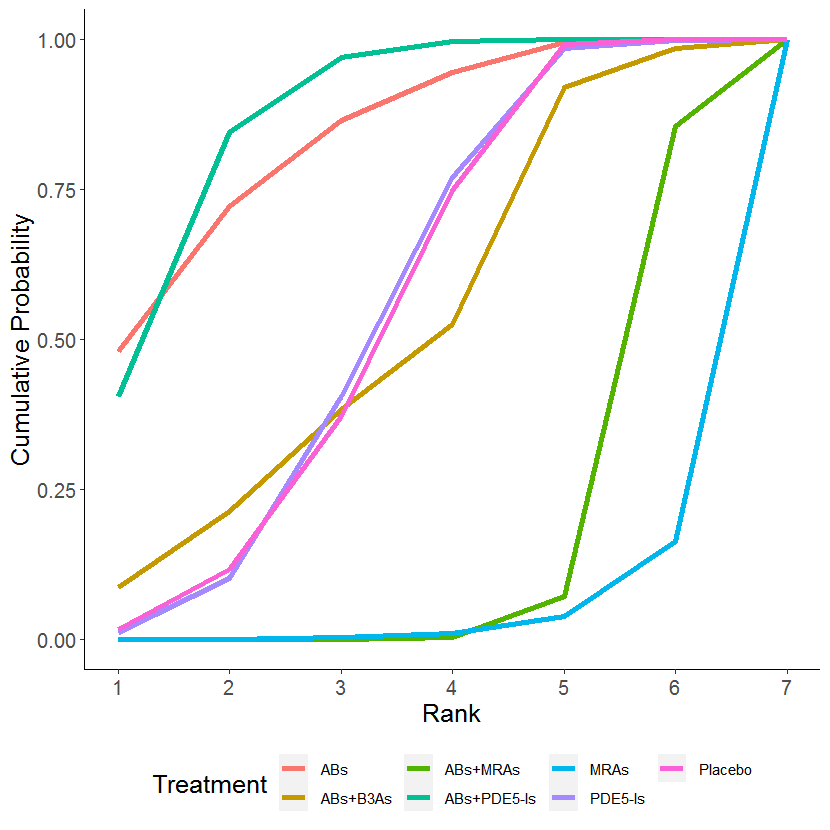
**

**For the subgroup PVR with treatment time ＜12W, the original research is less, and the network connection diagram has not been formed, so it cannot be analyzed.**
